# Bronchoplasty for the treatment of carcinoid tumour

**DOI:** 10.1002/rcr2.680

**Published:** 2020-12-03

**Authors:** Hazem Zribi, Ahmed Ben Ayed, Amina Abdelkbir, Imen Bouassida, Sarra Maazaoui, Adel Marghli

**Affiliations:** ^1^ Thoracic Surgery Department Abderrahmen Mami University Hospital Ariana Tunisia; ^2^ Pneumology PAV II Department Abderrahmen Mami University Hospital Ariana Tunisia

**Keywords:** Carcinoid tumour, lung neoplasm, sleeve, surgical resection

## Abstract

Lung carcinoid tumours are rare neoplasms with a favourable prognosis. Bronchoplasty can be a conservative treatment for typical carcinoid tumours and can be applied for patients with limited respiratory function. We report the case of a 34‐year‐old woman, with a polypoid tumour located at the distal right main bronchus. Bronchial biopsy showed a typical carcinoid tumour. After resection of the tumour in the right main bronchus, bronchoplasty was performed by end‐to‐end anastomosis of the remaining right main bronchus, right upper lobar bronchus, and the upper bronchus intermedius. Bronchoscopy showed a good quality anastomosis with slightly reduced endoluminal calibre only. The post‐operative period was uneventful and the patient was discharged at the seventh day. One year later, no complications occurred and the patient is still being followed up regularly.

## Graphical abstract

In this article, we try to demonstrate the possibility of creating a "neo‐spur" without lobectomy to treat an intra‐bronchial typical carcinoid tumor.

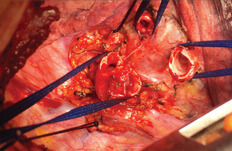

## Introduction

Lung carcinoid tumours are neuroendocrine tumours accounting for 0.4–3% of resected lung tumours. They are classified into typical and atypical carcinoid tumours according to the World Health Organization 2004 classification. Typical carcinoid tumours usually have a favourable prognosis after surgical resection alone [1].

Here, we describe a bronchoplasty procedure to resect the tumour without lobectomy or pneumonectomy.

## Case Report

A 34‐year‐old woman presented to the Pneumology Department with a one‐year history of haemoptysis.

**Figure 1:**
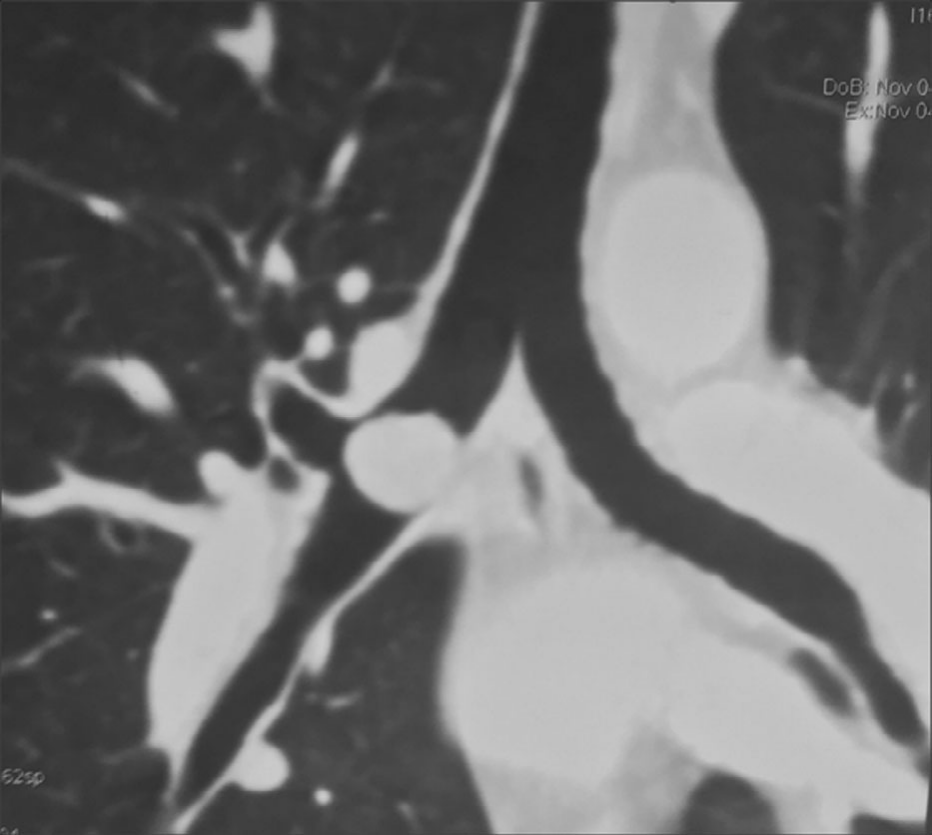
Computed tomography scan showed well‐circumscribed endoluminal formation in the posterior wall of primary right bronchus measuring 2 cm.

The clinical examination and the chest X‐ray were normal. Computed tomography (CT) scan showed well‐circumscribed endobronchial tumour in the posterior wall of the right bronchus measuring 2 cm (Fig. 1). Bronchoscopy showed an obstructive polypoid tumour in the distal right main bronchus arising from the posterior bronchial wall. The tumour bled easily on contact with the bronchoscope. Bronchial biopsy showed a typical carcinoid tumour.

Through a posterolateral right thoracotomy, exploration found a 2‐cm lesion located at the distal right main bronchus just in front of the upper lobe bronchial spur.

**Figure 2:**
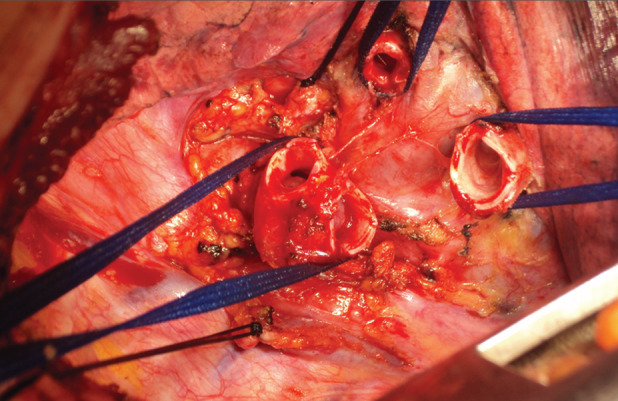
Resection of the primary right bronchus at 2 cm from the carina and carrying the upper lobar bronchus and intermediate bronchus.

After resection of the tumour in the right main bronchus, at 2 cm from the main carina, the upper lobar bronchus and intermediate bronchus were anastomosed with the remaining right main bronchus (Fig. 2), by a running PDS® 4/0 suture. The bronchial suture was covered by mediastinal pleura followed by a lymph node dissection. Pathology of the resected specimen showed a carcinoid tumour without lymph node involvement. Bronchoscopy performed at the end of the procedure showed a good quality anastomosis with less than 20% narrowing of the airway.

The post‐operative period was uneventful and the patient was discharged on the seventh day after a bronchoscopy showed the same results as the previous one.

The final stage of the tumour was pT1aN0MX.

One year later, no complications occurred: CT scan did not show any change in the airway calibre and no evidence of tumour recurrence. The patient is still being followed up regularly.

## Discussion

Lung carcinoid tumour is a well‐differentiated neuroendocrine neoplasm accounting for approximately 2% of pulmonary lung tumours. The majority of them are located in the bronchus with only 10% in the lung parenchyma. Occasionally, they are located in the main carina or trachea [2]. This tumour affects patients more frequently in the fifth decade. Bronchopulmonary carcinoid tumours occur more frequently in women than in men. Bronchial carcinoids are usually diagnosed by bronchoscopy and biopsy.

After surgical treatment, typical carcinoid tumours have a favourable prognosis compared to other lung neoplasm. Recurrence or metastatic disease occurs in 2–11% [3].

Surgery offers curative treatment. If surgical resection cannot be performed due to poor cardiopulmonary status, tumours without cartilaginous extension can be removed by endobronchial therapy [4].

Bronchoplastic resection for lung cancer is typically indicated for tumours located in bronchial orifice [5]. The American College of Chest Physicians recommends a sleeve resection or bronchoplasty procedure over a pneumonectomy for central Stage I non‐small cell lung cancer [6]. Indeed, limited parenchyma resections are preferred for a typical carcinoid neoplasm [5, 6] and radical surgery is reserved for atypical cases [6]. In our case, we preferred not to sacrifice the upper right lobe and to make a “neo‐spur”‐primary bronchus anastomosis. Lymph node involvement is an important prognostic factor. Thus, lymph node dissection is essential after the resection of the tumour [4, 6].

Bronchial anastomosis can be made using various techniques. In a recent study, Palade et al. [4] found no significant difference between using telescoping continuous anastomosis and interrupted suture without telescoping. Currently, there is no evidence to favour one anastomotic or suture technique over another [5]. In our practice, we use a running suture without telescoping. Making a neo‐spur is technically challenging because it uses a “double anastomosis”, the first one between the upper right lobe bronchus and the intermediate bronchus, and the second with primary right main bronchus.

Bronchial anastomosis can be complicated by bronchopleural fistula, dehiscence, ischaemia, and stenosis. Thus, we continue to follow‐up our patient who had an uneventful post‐operative period until the date of writing this article.

Neo‐spur creating technique can be a conservative treatment for typical carcinoid tumours with good result. It can be applied for patients with limited respiratory function.

### Disclosure Statement

Appropriate written informed consent was obtained for publication of this case report and accompanying images.
